# *Toxoplasma gondii* Type I TR and ROP16 Synergistically Downregulate IL-12 to Inhibit Host Reactive Oxygen Species Production

**DOI:** 10.3390/pathogens14020171

**Published:** 2025-02-08

**Authors:** Xiaoling Geng, Ruifang Li, Jingying Du, Manyu Zhang, Wei Jiang, Qing Sun, Rongsheng Mi, Shuang Qin, Quan Wang

**Affiliations:** 1 Shanghai Veterinary Research Institute, Chinese Academy of Agricultural Science, Shanghai 200241, China; gxl20240213@163.com (X.G.); liruifang107@163.com (R.L.); ddujingying@163.com (J.D.); zhangmy301237@163.com (M.Z.); jiangweijw99@163.com (W.J.); sun7355608520@163.com (Q.S.); rongshengmi@shvri.ac.cn (R.M.); qinshuang202309@163.com (S.Q.); 2 College of Veterinary Medicine, Northwest A&F University, Yangling 712100, China

**Keywords:** *Toxoplasma gondii*, CRISPR/Cas9, thioredoxin reductase, rhoptry protein 16, immune escape, oxidative stress

## Abstract

*Toxoplasma gondii* is an obligate intracellular opportunistic protozoan parasite. *T. gondii* invasion disturbs the balance between reactive oxygen species (ROS) production and antioxidant capacity in the host, triggering the oxidative stress response. Thioredoxin reductase (TR) of *T. gondii* helps to escape ROS-induced damage in the host, whereas *T. gondii* rhoptry protein 16 (ROP16) downregulates host innate immunity to suppress excessive inflammation and inhibit ROS production. However, whether TR and ROP16 synergistically promote resistance to ROS-induced damage remains unclear. Here, we used the CRISPR/Cas9 technology to successfully obtain a double *TR* and *ROP16* gene knockout *T. gondii* strain. The double deletion of *TR* and *ROP16* in *T. gondii* weakened its growth ability in vitro and decreased its virulence in vivo. Moreover, the double deletion of *TR* and *ROP16* resulted in a lower antioxidant capacity, higher degree of lipid oxidation, and elevated ROS levels in the parasite and host cells. Interestingly, the deletion of the *TR* and *ROP16* genes in *T. gondii* synergistically increased IL-12 levels, which triggered host cells to produce more ROS to resist *T. gondii* infection. These results show that TR and ROP16 in *T. gondii* play a synergistic role, facilitating resistance to ROS damage incurred by host immune cells through different pathways.

## 1. Introduction

*Toxoplasma gondii*, an intracellular parasite of the phylum *Apicomplexa*, class *Conoidasida*, and order *Eucoccidiorida*, is a feline intestinal coccidium species. *T. gondii* shows obligate parasitism in the nucleated cells of the host and causes toxoplasmosis, a common parasitic disease in nearly one-third of the world’s population [1,2]. When a host is infected with *T. gondii*, it develops systemic clinical symptoms, especially when its immune system is weakened [3]. Typical clinical manifestations of toxoplasmosis include toxoplasmic encephalitis, anterior/posterior uveitis, and neurological, ocular, or systemic damage in neonates [4]. Infection of animals, e.g., sheep and goats, with *T. gondii* may result in fetal death, mummification, abortion, stillbirths and neonatal deaths, which cause serious economic losses [5]. Therefore, *T. gondii* causes serious harm to human health and the livestock industry [6].

Innate immunity is the first line of host defense against *T. gondii* infection. The immune effector cells release reactive oxygen species (ROS) following the activation of the IL-12-IFNγ-STAT1 pathway to eliminate *T. gondii* infection [7,8,9]. The immune microenvironment of the host is toxic to *T. gondii* [10]. The virulence factors of *T. gondii* help to counter ROS-induced damage in different ways [11]. On the one hand, *T. gondii* has developed an antioxidant defense system, comprising superoxide dismutase, catalase, glutathione peroxidase, and peroxiredoxins, which protect *T. gondii* cells against ROS [12]. *T. gondii* also uses the thiol-reduction system, including thioredoxin reductase (TR), glutathione, glutaredoxin, and a specific reductase to resist oxidative damage [12,13]. TR is a NADPH-dependent FAD domain-containing dimeric selenium enzyme belonging to the family of pyridine nucleotide–disulfide oxidoreductases, which includes TrxR1, TrxR2, and TrxR3. The main function of these proteins is to resist oxidative damage brought about by the immune system of the host and maintain the reduced form of thioredoxin (Trx) by using NADPH [14,15,16,17]. On the other hand, *T. gondii* inhibits the production of ROS in host immune cells by downregulating the IL-12-IFN-γ-STAT signaling pathway. STAT3/STAT6 are activated and phosphorylated by rhoptry protein 16 (ROP16) secreted by type I *T. gondii*, which ultimately downregulates the activity of the IL-12-IFNγ-STAT1 pathway, thereby reducing the production of ROS and resisting the oxidative stress damage inflicted by the host [18,19,20,21]. However, whether ROP16 and TR secreted by *T. gondii* have a synergistic effect, promoting resistance to oxidative damage, is currently unknown.

To date, no protein has been found to have a synergistic effect with TR in resisting ROS damage from host immune cells. In the present study, we used CRISPR/Cas9 technology to knock out *ROP16* in the *TR* knockout strain of *T. gondii* to obtain the *TR*-*ROP16*-KO strain. To investigate the functional contributions of TR and ROP16, we detected and analyzed the factors related to the ROS-scavenging pathway of *T. gondii*.

## 2. Materials and Methods

### 2.1. T. gondii and Cell Cultures

Tachyzoites of the *T. gondii* strain RH, *TR* gene deletion strain (*TR*-KO), and *ROP16* gene deletion strain (*ROP16*-KO) were maintained in vitro by passaging in African green monkey kidney Vero cells or the mouse macrophage cell line RAW264.7. Vero and RAW264.7 cells were grown in T75 cell culture flasks containing Dulbecco’s Modified Eagle Medium (DMEM) supplemented with 10% fetal bovine serum, 2 mM glutamine, 100 kU/L streptomycin, and 400 kU/L penicillin, at 37 °C in the atmosphere of 95% air and 5% CO_2_. The infected Vero cells were lysed through a 5-gauge needle, and the tachyzoites were filtered using a 5 µm pore size Millipore filter (Merck-Millipore, Darmstadt, Germany). The purified parasites were counted using a cell counting chamber under a microscope (Olympus CKX53, Tokyo, Japan).

### 2.2. Construction of Transgenic Parasite Strains

All plasmids, gRNAs, and primers used in this study are listed in Supplementary Table S1. A double gene knockout strain (*TR*-*ROP16*-KO) was constructed using the CRISPR/Cas9-mediated gene targeting technology. In this study, to ensure *ROP16* gene knockout, a CRISPR/Cas9 vector expressing dual-guide RNA was constructed. First, full-length sequences of the *ROP16* gene were downloaded from the NCBI website (GenBank: GQ249080.1), and gRNA1 and gRNA2 were designed using an optimized CRISPR design algorithm (http://chopchop.cbu.uib.no). Next, guide RNA targeting the ROP16 gene locus was used to replace the single-guide RNA site in the pSAG1::CAS9-U6:sgUPRT plasmid (#54467, Addgene, https://www.addgene.org/) using a ClonExpress MultiS one-step cloning kit (Vazyme Biotech, Nanjing, China) to construct the plasmids pSAG1:CAS9-U6:sgROP16-1 and pSAG1:CAS9-U6:sgROP16-2. Finally, the pSAG1::CAS9-U6:sgROP16 plasmid containing dual gRNAs was obtained by ligating the pSAG1::CAS9-U6::sgROP16-1 plasmid and gRNA2 fragment digested with the *Kpn*I and *Xho*I enzymes (Takara, Kyoto, Japan) using a T4 DNA Ligation Kit Ver.2.1 (Takara, Kyoto, Japan). To construct the *TR*-*ROP16*-KO strain, the pROP16::CAT-D plasmid was constructed by inserting the 3′ and 5′ regions flanking the ROP16 gene amplified from the genomic DNA of the RH strain into both sides of the CAT resistance fragment of the pCas9-CAT plasmid. Chloramphenicol (CAT) formed the CAT* cassette for positive screening, which was cloned from the pCas9-CAT plasmid (#80323, Addgene, https://www.addgene.org/). The pSAG1:CAS9::TgU6:sgROP16 and pROP16::CAT-D plasmids (Supplementary Figure S1) were transferred into the tachyzoites of the *TR*-KO strain by electroporation, and homologous recombination in the parasites was confirmed by PCR.

### 2.3. Western Blot Analysis

Freshly purified tachyzoites (1 × 10^8^) were lysed using 200 μL of protein lysate (a mixture of the radioimmunoprecipitation assay buffer and phenylmethylsulfonyl fluoride at a 100:1 ratio) on ice for 3–5 min, and the supernatant of the sample was obtained by centrifugation. Equivalent amounts of the sample supernatant were electrophoresed on 10% SDS polyacrylamide gels and then electroblotted onto 0.45 µm nitrocellulose membranes. Mouse anti-ROP16 (1:1000) and rabbit anti-TR (1:2000), which were prepared and preserved in our laboratory previously, were used as primary antibodies. Horseradish peroxidase-labeled goat anti-mouse IgG and goat anti-rabbit IgG (1:4000) were used as secondary antibodies (Bersee, Beijing, China). Membranes were visualized using a standard enhanced chemiluminescence system (Bio-Rad, Hercules, CA, USA).

### 2.4. Immunofluorescence Assay

Freshly purified tachyzoites (1 × 10^5^) were used to infect Vero cells seeded on coverslips for 4 h. Vero cells were washed with phosphate-buffered saline (PBS) to remove parasites that failed to invade the cells. Subsequently, the samples were further cultured for 20 h at 37 °C in an atmosphere of 95% air and 5% CO_2_. Cell samples were fixed with 4% paraformaldehyde and permeabilized with 0.5% Triton X-100 (Licheng Biology, Nanjing, China). Subsequently, the samples were incubated with anti-ROP16 (1:1000) and rabbit anti-TR (1:2000) primary antibodies for 1 h at 37 °C, and then Alexa Fluor 488- and Alexa Fluor 594-labeled IgG secondary antibodies (Merck, Rahwa, NJ, USA) for 1 h at 37 °C. The nucleus was visualized by incubation with DAPI for 15 min at room temperature. The cells were observed under a fluorescence microscope (LSM 900, Zeiss, Oberkochen, Germany).

### 2.5. Invasion and Proliferation Assays

For the invasion assays, freshly purified tachyzoites (1 × 10^6^) were labeled using a CDFA-SE cell proliferation kit (Beyotime, Shanghai, China). The labeled tachyzoites were inoculated into each well of 6-well plates containing Vero cells at 90% confluency and cultured for 12 h at 37 °C in an atmosphere of 95% air and 5% CO_2_. The wells were washed with blank DMEM to remove parasites that did not invade Vero cells. Then, the cells were completely digested by trypsin, resuspended in 500 μL of DMEM supplemented with 10% fetal bovine serum, and analyzed with a Cytomics FC 500 flow cytometer (Beckman Coulter, Brea, CA, USA). The percentage of cells infected with *T. gondii* was calculated using FlowJo software version 7.6.1 (Tree Star Inc., Ashland, OR, USA).

For proliferation assays, freshly purified tachyzoites (1 × 10^5^) were inoculated into each well of 6-well plates containing Vero cells at 80% confluency and cultured for 4 h at 37 °C. The wells were washed with PBS to remove parasites that failed to invade Vero cells. Then, the cells were cultured at 37 °C in an atmosphere of 95% air and 5% CO_2_ for 24 h. One hundred vacuoles of *T. gondii* were randomly counted, and the number of tachyzoites in each vacuole was recorded. The results were expressed as the percentage of vacuoles containing different numbers of tachyzoites.

### 2.6. Plaque Assay

A monolayer of Vero cells grown in 6-well plates was infected with 200 freshly purified tachyzoites per well for 6 days. The monolayers were then fixed with 4% paraformaldehyde (Labgic Technology Co., Ltd., Beijing, China) at 4 °C for 20 min, stained with crystal violet for 15 min, and gently washed thrice with PBS. Plaques formed by the growing parasites were counted using an inverted microscope (Olympus CKX53, Tokyo, Japan). All strains were tested thrice, each with three technical replicates.

### 2.7. Virulence Assay

Freshly purified tachyzoites (1 × 10^3^) were inoculated into the abdominal cavity of 7-week-old female KunMing mice (JieSiJie, Shanghai, China), and animals injected with the same volume of sterile saline were used as the blank group. The time to death of the mice inoculated with different strains of *T. gondii* was monitored. The experimental results were visualized by plotting the survival curves using Prism 8.0 (GraphPad Software Inc., La Jolla, CA, USA).

### 2.8. Determination of Malondialdehyde (MDA), Total Antioxidant Capacity (T-AOC), and Reactive Oxygen Species (ROS) in Tachyzoites

MDA, T-AOC, and ROS levels were determined in freshly purified tachyzoites of *T. gondii* RH, TR-KO, ROP16-KO, and TR-ROP16-KO strains. To measure MDA levels, tachyzoites (1 × 10^8^) were lysed with 200 μL of IP (Beyotime, Shanghai, China) cell lysate, and after the supernatant was collected, MDA levels were determined at 535 nm with an MDA assay kit (Beyotime, Shanghai, China) and expressed in MDA amount per milligram of protein (μmol/mg). To measure T-AOC levels, tachyzoites (1 × 10^8^) were resuspended in 2 mL of PBS. The mixed solution was sonicated on ice, the supernatant was collected, and T-AOC levels were measured at 593 nm using a T-AOC assay kit (Beyotime, Shanghai, China). The total antioxidant capacity results were represented as the concentration of the FeSO4 standard solution (mM). To determine the ROS levels, tachyzoites (1 × 10^7^) were labeled with 2′,7′-dichlorofluorescein diacetate (Beyotime, Shanghai, China) at 37 °C for 20 min and resuspended in 200 μL of DMEM. The resuspended solution was added to a 96-well plate, and the intracellular fluorescence intensity was determined using a BioTek Synergy 2 luminescence microplate reader (BioTek, Winooski, VT, USA). Intracellular ROS levels were expressed as multiples of those in the RH strain.

### 2.9. Determination of ROS Levels in Macrophages

Freshly purified tachyzoites (1 × 10^6^) were inoculated into a 6-well plate containing RAW 264.7 cells at 80% confluency and cultured for 2, 4, 6, or 12 h at 37 °C in an atmosphere of 95% air and 5% CO_2_. The wells were washed with DMEM to remove *T. gondii* that failed to invade RAW 264.7 cells. Then, the cells were digested by trypsin, labeled with 2′,7′-dichlorofluorescein diacetate (Beyotime, Shanghai, China) at 37 °C for 20 min, and washed thrice with DMEM, and the ROS levels were measured using a BioTek Synergy 2 luminescence fluorescence microplate reader (BioTek, Winooski, VT, USA). The results were expressed as multiples of the fluorescence intensity relative to that of the blank control group.

### 2.10. Quantitative Reverse Transcriptase PCR

Total RNA was extracted from macrophages infected with tachyzoites of *T. gondii* RH, TR-KO, ROP16-KO, and TR-ROP16-KO strains, and cDNA was obtained using a reverse transcription kit (Takara, Kyoto, Japan). Quantitative detection of IL-12 mRNA in macrophages was performed with a SYBR-Green Premix Ex Taq kit (Takara, Kyoto, Japan). The expression level of the *β-actin* gene was used as a reference. The primers used in RT-qPCR are listed in Supplementary Table S2; relative fluorescence quantitative PCR (Applied Biosystems 7500 Real-Time PCR System, Thermo Fisher, Waltham, MA, USA) was used to detect the mRNA levels of IL-12. Three biological replicates were used in this study. The mRNA transcription level of each cytokine gene was analyzed using the 2^−ΔΔCt^ method.

### 2.11. Determination of Mouse Serum IL-12 Levels

Tachyzoites (5 × 10^6^) of *T. gondii* RH, *TR*-KO, *ROP16*-KO, and *TR*-*ROP16*-KO strains were inoculated into the abdominal cavity of 6-week-old female Kunming mice (JieSiJie, Shanghai, China). Blood was collected at 0.5, 2, 4, and 6 h after the infection with tachyzoites via tail-vein bleed. Samples were centrifuged at 2000 rpm for 8 min, and serum was collected and stored at −80 °C. The serum level of IL-12 was determined by an ELISA kit (Biolegend, San Diego, CA, USA). Three biological and three technical replicates were used in this experiment.

### 2.12. Statistical Analysis

SPSS (version 17.0; SPSS, Inc., Chicago, IL, USA) was used for all analyses. Data were analyzed using one-way analysis of variance to determine the statistical significance of strain effects on various parameters. The results were expressed as the mean ± standard deviation. Effects were considered as significant if *p* < 0.05 and extremely significant if *p* < 0.01.

## 3. Results

### 3.1. Construction of the TR and ROP16 Double Gene Knockout Strain Using the CRISPR/Cas9 Method

**Figure 1:**
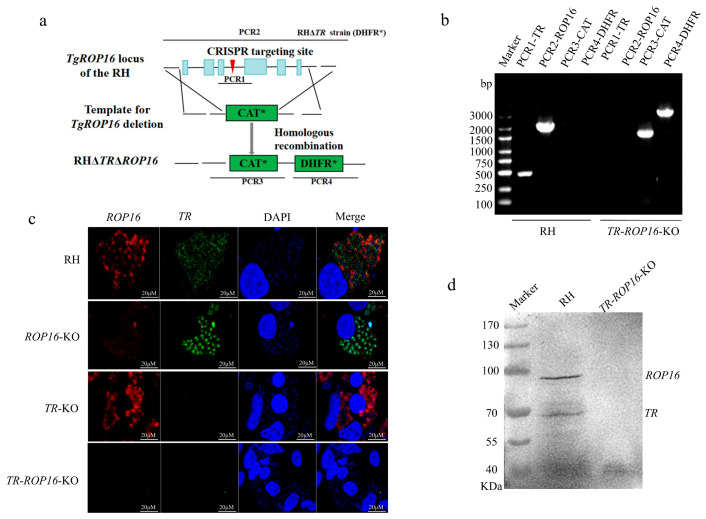
Construction of the *TR*-*ROP16*-KO strain of *T. gondii* type I by using CRISPR-Cas9 technology. (**a**) Schematic representation of the CRISPR-Cas9 system used for disrupting the ROP16 gene by insertion of the CAT*-Ts cassette. (**b**) PCR validation of *ROP16* gene deletion in the monoclonal strain. (**c**) Identification of the *TR* and *ROP16* deletion mutant by immunofluorescence. (**d**) Identification of the *TR* and *ROP16* deletion mutant by Western blot.

To investigate the biological roles of *TR* and *ROP16* in the *T. gondii* type I strain RH, we used the CRISPR/CAS9 technique to disrupt the *ROP16* gene in the *TR*-KO strain by inserting the chloramphenicol resistance fragment (CAT*-Ts) in the sgRNA-targeted coding region, enabling pROP16-CAT-D to be used for the deletion of the entire ROP16-coding sequence (Figure 1a). Single-clone strains were identified using PCR, and the results showed that the CAT coding sequence was successfully inserted into the target position (Figure 1b). To further confirm that the *TR* and *ROP16* genes of *T. gondii* were both deleted, immunofluorescence and Western blot analyses were used to show the absence of TR and ROP16 protein expression in the *TR*-*ROP16*-KO strain (Figure 1c,d). These results were consistent with the PCR results, proving that the *TR*-*ROP16*-KO strain of *T. gondii* was successfully obtained.

### 3.2. Double Deletion of the TR and ROP16 Genes in T. gondii Decreases Growth Capacity In Vitro

**Figure 2:**
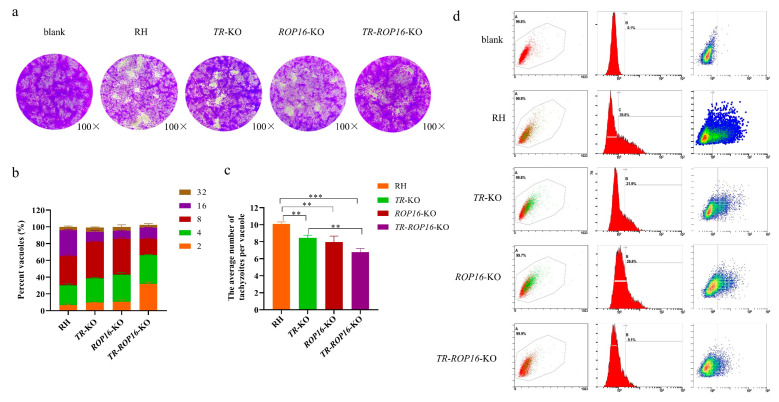
Effects of *TR* and *ROP16* deletion on the proliferation and invasion of *T. gondii.* (**a**) Plaque assay of the parental RH strain, *TR*-KO, *ROP16*-KO strain, and *TR*-*ROP16*-KO strain over a 7-day infection of Vero cells. (**b**,**c**) The effects of *TR* and *ROP16* deletion on proliferation in vitro. Data are expressed as the percentage of vacuoles containing 2, 4, 8, 16, and 32 parasites: ** *p* < 0.01; *** *p* < 0.001. (**d**) Flow cytometry results of *TR* and *ROP16* deletion strains.

To investigate whether the double gene deletion of *TR* and *ROP16* affected the growth of *T. gondii* RH strain in vitro, plaque assays were performed on the monolayers of Vero cells in six-well plates, and the number and size of the plaques were analyzed. Compared with the data for the RH or single-gene deletion strains, the growth of the *TR*-*ROP16*-KO strain was significantly reduced (Figure 2a). To assess the proliferative ability of the *TR*-*ROP16*-KO strain in vitro, we measured the number of tachyzoites in parasitophorous vacuoles at 36 h after infection. The *TR*-*ROP16*-KO strain had significantly fewer tachyzoites than other strains (Figure 2b,c). Next, to evaluate the invasive ability of the *TR*-*ROP16*-KO strain in vitro, the parasites were labeled with a fluorescent probe, and the flow cytometry results indicated that the invasion rate of the *TR*-*ROP16*-KO strain was remarkably decreased (Figure 2d). These results implied that the double deletion of *TR* and *ROP16* affected the growth of *T. gondii*.

### 3.3. Double Deletion of the TR and ROP16 Genes Changes T. gondii Virulence in Mice

**Figure 3:**
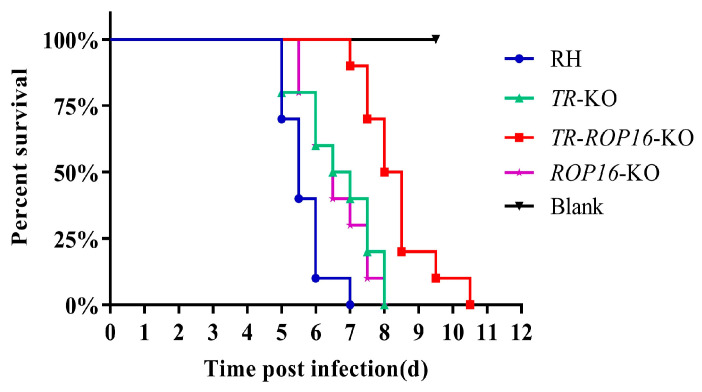
Effect of *TR* and *ROP16* deletion strains on the virulence of *T. gondii*. Ten KunMing mice were infected with 100 RH, *TR*-KO, *ROP16*-KO, and *TR*-*ROP16*-KO tachyzoites, and their survival was assessed over 15 days.

To evaluate the role of *TR* and *ROP16* in the virulence of *T. gondii*, 1000 tachyzoites of the *TR*-*ROP16*-KO, *TR*-KO, *ROP16*-KO, and RH strains were injected intraperitoneally into Kunming mice. The mice infected with the RH strain died within 7 days and those infected with the *TR*-KO or *ROP16*-KO strain died within 8 days. However, mice infected with the *TR*-*ROP16*-KO strain survived for significantly longer and died within 10.5 days (Figure 3). These results indicate that the double deletion of *TR* and *ROP16* further reduces the virulence of type I *T. gondii*.

### 3.4. Double Deletion of the TR and ROP16 Genes in T. gondii Increases Intracellular Oxidative Stress Response

**Figure 4:**
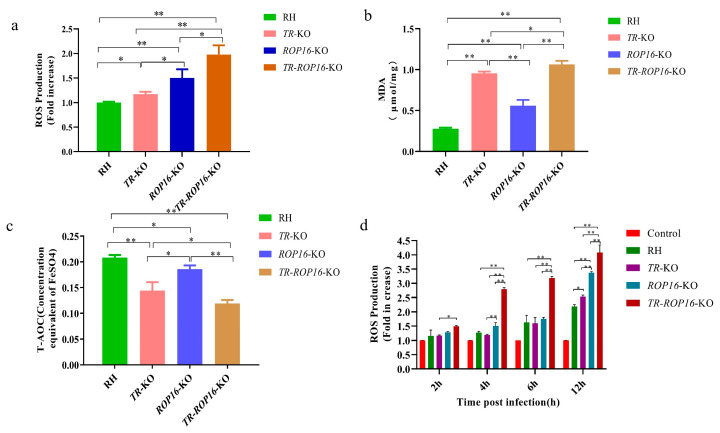
Effect of *TR* and *ROP16* deletion on oxidative stress of *T. gondii.* (**a**) ROS levels of different deletion strains. (**b**) MDA levels of different deletion strains. (**c**) T-AOC levels of different deletion strains. The RH strain was used as the control. The results of the tested group are expressed as a multiple of the control group. All samples were measured in triplicate. (**d**) Effects of *TR* and *ROP16* deletion of *T. gondii* on ROS in mouse macrophages after infection. The RAW264.7 cells were used as the control. The results are expressed as fold increases in fluorescence density in the test group as compared with the control group. All samples were measured in triplicate. The results were presented as mean ± SD (* *p* < 0.05, ** *p* < 0.01).

To test whether the double gene deletion of *TR* and *ROP16* affected the production of intracellular ROS, the levels of oxidative stress in the parasites were determined by measuring MDA, T-AOC, and ROS. In parasites of the *TR*-*ROP16*-KO strain, we observed an increased accumulation of ROS (Figure 4a), and the MDA level was significantly higher (Figure 4b), whereas the T-AOC level was significantly lower compared with those in the *TR*-KO, *ROP16*-KO, and RH strains (Figure 4c). In addition, to explore whether the deletion of the *TR* and *ROP16* genes in *T. gondii* affected the oxidative stress response of host cells, we evaluated ROS levels in mouse macrophages infected with different *T. gondii* strains. *T. gondii* infection elevated reactive ROS levels in macrophages in a steep, time-dependent fashion. Intriguingly, the rate of increase in ROS levels in cells infected with the *ROP16*-KO strain was higher than that in cells infected with the *TR*-KO strain. Moreover, ROS levels in cells infected with the *TR*-*ROP16*-KO strain were significantly higher than those in cells infected with the single-gene deletion strains (Figure 4d). These studies suggest that the double deletion of the *TR* and *ROP16* genes in *T. gondii* synergistically leads to an increase in oxidative stress levels in host cells.

### 3.5. Cytokine Changes in the Host Are Affected by the Deletion of the TR and ROP16 Genes in T. gondii

**Figure 5:**
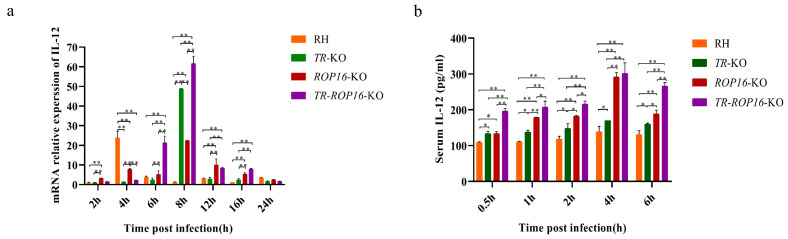
Effects of *TR* and *ROP16* deletion of *T. gondii* on cytokines in innate immune signal pathway. (**a**) The mRNA transcription level of IL-12 in mouse macrophages after infection with different deletion strains of *T. gondii*. (**b**) The expression of IL-12 in the serum of mice infected with different deletion strains of *T. gondii* (pg/mL). Each sample was performed in triplicate. The RH strain was used as the control. The results of the test group are expressed as the mRNA relative expression level. The results were presented as mean ± SD (* *p* < 0.05, ** *p* < 0.01).

Cytokines play an important role in the body reaction against *T. gondii* infections. To address whether the double gene deletion of *TR* and *ROP16* affected the production of cytokines in host cells, RAW264.7, cells were infected with tachyzoites of the RH, *TR*-KO, *ROP16*-KO, or *TR*-*ROP16*-KO strain for at 2, 4, 6, 8, 12, and 24 h. Cytokine expression levels in mouse macrophages at different time points of infection were detected by RT-qPCR. We observed that *T. gondii* infection changed IL-12 mRNA levels in host cells. Notably, IL-12 mRNA levels in RAW264.7 cells inoculated with the *TR*-KO strain were significantly higher than those in the cells inoculated with the *ROP16*-KO strain at 8 h after infection (Figure 5a). Interestingly, IL-12 mRNA levels in RAW264.7 cells infected with the *TR*-*ROP16*-KO strain were significantly increased at 6 to 8 h after infection, compared with IL-12 expression levels in cells inoculated with other strains (Figure 5a). In addition, to determine whether the double deletion of the *TR* and *ROP16* genes affected IL-12 production in the host, the mice were infected with tachyzoites of the RH, *TR*-KO, *ROP16*-KO, or *TR-ROP16*-KO strains, and serum samples were obtained at 0.5, 1, 2, 4, and 6 h post infection for IL-12 detection by ELISA. We found that at 0.5–6 h after infection, IL-12 levels in the mice infected with the *TR*-KO, *ROP16*-KO, and *TR*-*ROP16*-KO tachyzoites were consistently higher than those in the control group at every detection time point. Furthermore, serum levels of IL-12 in mice infected with *TR*-*ROP16*-KO tachyzoites were significantly higher than those in mice from the *TR*-KO- and *ROP16*-KO-infected groups (Figure 5b). Surprisingly, during the infection period, IL-12 levels in mice infected with the *TR*-*ROP16*-KO strain were increased not only due to the absence of *ROP16*, but also because of the *TR* deficiency.

## 4. Discussion

The effector factors *ROP16* and *TR* of *T. gondii* play pivotal roles in the protection against host innate immunity and directly affect the consequences of infection and disease development [17,22]. During invasion into host cells, ROP16 is secreted from the rhoptries of *T. gondii* and rapidly localizes to the host cell nucleus. [23]. Previous studies found that although ROP16 is polymorphic and virulence-dependent [24], deleting the *ROP16* gene in the type I strain affected the virulence of the parasite in mice [25], which was consistent with the results of our previous studies. Moreover, preliminary studies in our laboratory also showed that the invasion and proliferation abilities of the *ROP16*-KO type I strain were weaker than those of the type I RH strain. Furthermore, TR is a key secretory factor protecting the type I strain against host oxidative damage, as it reduces superoxide radicals through a direct redox reaction [26,27]. Deletion of the *TR* gene in the type I strain likely inevitably dysregulated the redox system. Preliminary studies in our laboratory showed that the invasion, proliferation, and virulence of the type I *TR*-KO strain were weaker than those of the RH and *TR* gene-complementary strains (*TR*-CO) [17]. However, the biological characteristics of the *TR*-*ROP16*-KO type I *T. gondii* strain devoid of both *ROP16* and *TR* remained unknown. Therefore, we examined the invasiveness, proliferation, and virulence of the *TR*-*ROP16*-KO strain using the same method. By using flow cytometry and microscopy, we established that the invasive and proliferative abilities of the *TR*-*ROP16*-KO strain were significantly lower than those of the *TR*-KO, *ROP16*-KO, and RH strains. The data on the survival of infected mice showed that the virulence of the *TR*-*ROP16*-KO strain was significantly lower than that of either of the single-gene deletion strains. These results indicate that the *TR* and *ROP16* genes in *T. gondii* are crucial for maintaining the biological activity of the parasite.

Oxidative stress is mainly caused by an imbalance between ROS production and antioxidant capacity of host cells [28]. Animal cells produce lipid oxides, such as MDA, which is a marker of oxidative stress [29,30]. In addition, cells have various antioxidants that eliminate various ROS produced in the body to protect against oxidative stress [31]. Therefore, the level of oxidative stress can be assessed by measuring the levels of MDA, T-AOC, and ROS in parasite cells. Our previous research showed that the oxidative stress level in the *TR*-KO strain of *T. gondii* was significantly higher than that in the wild and *TR*-CO strains, indicating that *TR* gene deletion affected the antioxidant capacity of *T. gondii* [17]. Our present results showed that the extent of oxidative stress in the *TR*-*ROP16*-KO strain was significantly higher than those of the wild RH strain as well as of the *TR*-KO and *ROP16*-KO strains. Specifically, the *TR*-*ROP16*-KO strain had the lowest antioxidant capacity, highest degree of lipid oxidation, and highest ROS level caused by parasites. This suggests that the *TR* and *ROP16* genes of *T. gondii* may play a synergistic role in resisting ROS damage brought about by host immune cells.

ROS, including H_2_O_2_, superoxide anion, and hydroxyl free radicals, are the byproducts of cellular oxygen metabolism [32,33]. Host immune cells (neutrophils, eosinophils, and macrophages) resist microbial infections by releasing ROS [34,35]. In addition, ROS produced by host innate immune response inhibit the activity of *T. gondii* in monocytes and production of IFN-γ in macrophages of infected mice [36]. In this study, ROS levels in macrophages inoculated with the *TR*-*ROP16*-KO, *TR*-KO, *ROP16*-KO, and RH strains were significantly higher than those in the control group, and the highest levels were observed in the cells infected by the *TR*-*ROP16*-KO strain between 4 and 12 h post infection. Therefore, we speculated that the high ROS levels observed in macrophages infected with *T. gondii* strains developed on the wild RH strain background may be caused by the TLR-induced MYD88-IL-12 pathway activation [37]. However, the dramatically increased ROS generation in macrophages inoculated with the *TR*-*ROP16*-KO strain may be owing to the deletion of the *TR* gene of *T. gondii*, which affected the release of antioxidant substances and conferred an inability to completely eliminate all types of ROS in cells. In addition, the deletion of the *ROP16* gene in *T. gondii* resulted in the accumulation of IL-12 in the host innate immune IL-12-IFN-γ-STAT1 signaling pathway, which indirectly affected ROS levels.

Cytokines play an important role in resistance to *T. gondii* infection, and immune effector cells trigger the release of ROS through the IL-12-IFNγ-STAT1 pathway to clear the infection of *T. gondii* [38,39,40]. Previous reports have indicated that ROP16 in *T. gondii* interferes with IL-12 levels in that signaling pathway to achieve parasitic reproduction in the host [41]. In this study, we found that IL-12 mRNA expression in RAW264.7 cells infected with the *ROP16*-KO strain was significantly increased at 6 to 8 h after infection, compared with that in cells infected with the RH strain. Moreover, ELISA showed that the IL-12 level in mice infected with *ROP16*-KO *T. gondii* was significantly higher than that in the RH strain. These results are consistent with those of the previous studies conducted in our laboratory. Similarly, TR helps resist the action of ROS produced by innate immunity [41]. TR is a component of the antioxidant system of *T. gondii* that catalyzes the conversion of Trx into its reduced form by consuming NADPH [42], which maintains the Trx redox state and resists free radical damage inflicted by host immune cells [43]. Surprisingly, compared with the effects of the RH strain, we found that IL-12 mRNA expression level in RAW264.7 cells infected with the *TR*-KO strain was significantly increased at 8 h after infection, and the serum level of IL-12 in mice infected with the *TR*-KO strain was also significantly elevated. To explain this finding, it is useful to remember that Trx (NADPH-dependent) in host cells takes part in the cytokine-induced denitrosylation and activation of NF-κB [44,45]. Therefore, we speculate that the deletion of the *TR* gene reduced the consumption of the NADPH substrate in host cells. In RAW264.7 cells infected with the *TR*-KO strain, a relatively sufficient level of NADPH maintained the activity of NF-κB and activated the production of IL-12, suggesting that TR downregulates IL-12 levels by inhibiting the NF-κB pathway. In addition, we found that IL-12 mRNA expression level in RAW264.7 cells infected with the *TR*-*ROP16*-KO strain was significantly increased at 6 to 8 h after infection, compared with the level in the cells infected by the RH, *TR*-KO, or *ROP16*-KO strains. Moreover, ELISA showed that IL-12 levels in mice infected with the *TR*-*ROP16*-KO strain were significantly higher than those in mice infected with other strains tested. These results suggest that the inflammatory response induced by the simultaneous deletion of *TR* and *ROP16* reached the highest degree, because ROP16 downregulated IL-12 levels through the phosphorylation of STAT3 or STAT6, and TR downregulated IL-12 levels through the inhibition of the NF-κB pathway. Regardless of whether *T. gondii* TR inhibits NF-κB by consuming host cell NADPH or not, based on the obtained results, we conclude that the deletion of the TR and ROP16 genes of *T. gondii* synergistically increased IL-12 levels and induced host cells to produce more ROS through the IL12-IFNγ-STAT1 signaling pathway.

## 5. Conclusions

In conclusion, we found that *ROP16* and *TR* are key virulence factors crucial for the growth of *T. gondii* in vitro and infectivity in vivo. The double deletion of *TR* and *ROP16* in *T. gondii* weakened the biological phenotype of the parasite. Interestingly, we found that the double deletion of *TR* and *ROP16* in *T. gondii* resulted in the lowest antioxidant capacity, highest degree of lipid oxidation, and highest ROS levels in the parasite and host cells. Importantly, we found that TR and ROP16 synergistically downregulated IL-12 levels, likely due to the facilitation of STAT3 or STAT6 phosphorylation by ROP16 and inhibition of the NF-κB pathway by TR. Therefore, the deletion of *TR* and *ROP16* genes of *T. gondii* synergistically increased IL-12 levels and augmented ROS production through the activation of the IL-12-IFNγ-STAT1 signaling pathway. This study clarifies the mechanism of the reduced virulence of the *TR*-*ROP16*-KO strain, providing a theoretical basis for the subsequent development of *T. gondii* vaccines as well as the prevention and control of toxoplasmosis.

## Data Availability

The original contributions presented in this study are included in the article; further inquiries can be directed to the corresponding author.

## Supplementary Materials

The following supporting information can be downloaded at: https://www.mdpi.com/article/10.3390/pathogens14020171/s1, Figure S1: Construction of pSAG1::CAS9-U6::sgROP16 and pROP16::CAT-D plasmids; Figure S2: The statistical analysis of the plaque number/size in the plaque assay; Table S1: Primer sequences used in the construction of TR-ROP16-KO strain; Table S2: Primer sequences used in RT-qPCR.
