# Supplementary material for: Toxoplasma gondii Type I TR and ROP16 Synergistically Downregulate IL-12 to Inhibit Host Reactive Oxygen Species Production
Source: Pathogens. 2025 Feb 8;14(2):171. doi: 10.3390/pathogens14020171 (PMC11858468; doi:10.3390/pathogens14020171)
Supplement: Supplementary file 1 [file pathogens-14-00171-s001.zip › Supplementary figures.pdf]

Supplementary Materials

Supplementary Figure S1

Figure S1

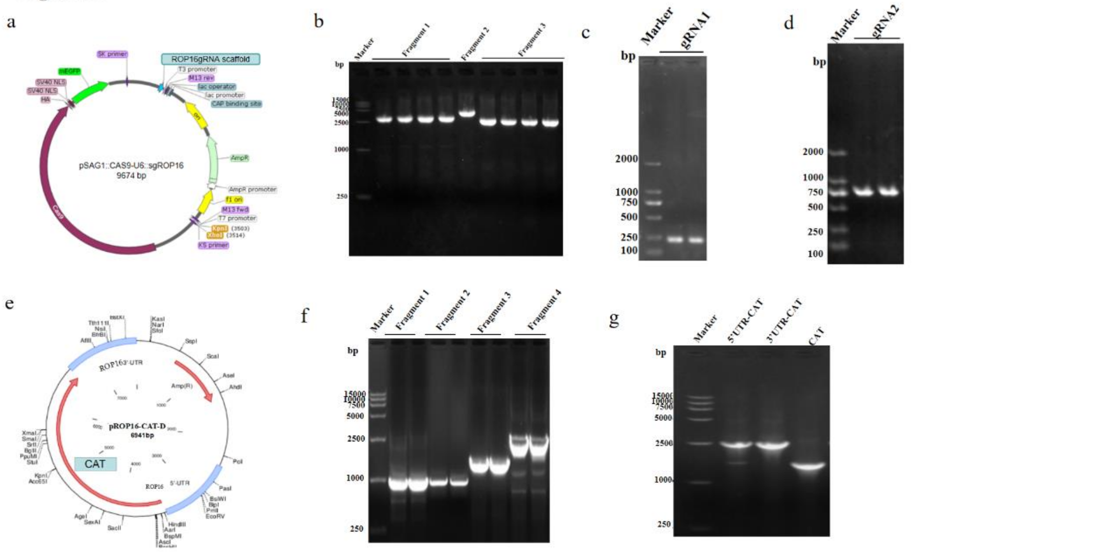

Figure S1. Construction of pSAG1::CAS9-U6::sgROP16 and pROP16::CAT-D plasmids

a. Plasmid map of pSAG1::CAS9 - U6::sgROP16

b. Amplification of pSAG1::CAS9-U6::sgROP16 plasmid fragments by PCR

c. Identify 220 bp of gRNA1 in pSAG1::CAS9-U6::sgROP16 plasmid

d. Identify 778 bp of gRNA2 in pSAG1::CAS9-U6::sgROP16 plasmid

e. Plasmid map of pROP16::CAT-D plasmid

f. Amplification of pROP16::CAT-D plasmid fragments by PCR

g. Identification of pROP16::CAT-D plasmid by PCR

Supplementary Figure S2

Figure S2

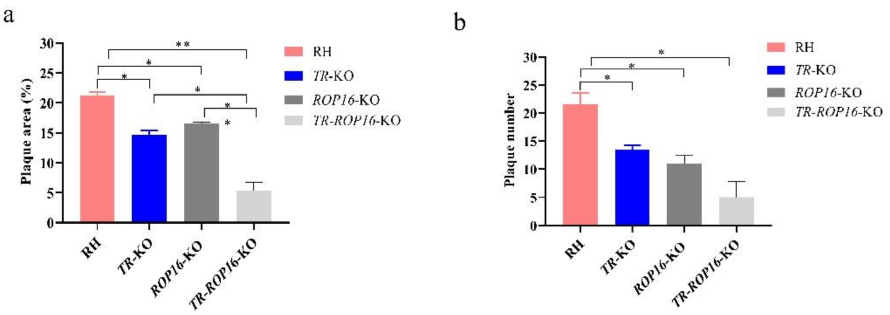

Figure S2. The statistical analysis of the plaque number/size in the plaque assay
